# *trans*-Bis(μ-2-hydroxy­ethanethiol­ato-κ^2^ *S*:*S*)bis­[dinitro­syliron(II)](*Fe*—*Fe*)

**DOI:** 10.1107/S1600536809048065

**Published:** 2009-11-18

**Authors:** Hon Man Lee, Show-Jen Chiou

**Affiliations:** a Department of Chemistry, National Changhua University of Education, Changhua, Taiwan 50058; b Department of Applied Chemistry, National Chiayi University, 300 Syuefu Road, Chiayi City 60004, Taiwan

## Abstract

The title complex, [Fe_2_(C_2_H_5_OS)_2_(NO)_4_], lies on a crystallographic inversion center. The Fe—Fe distance is characteristic of a metal–metal bond. In the crystal structure, inter­molecular O—H⋯O hydrogen bonds link complex mol­ecules into a two-dimensional network.

## Related literature

For iron–nitrosyl complexes, see: Chiang *et al.* (2004 ▶); Dillinger *et al.* (2007 ▶); Mazany *et al.* (1983 ▶).
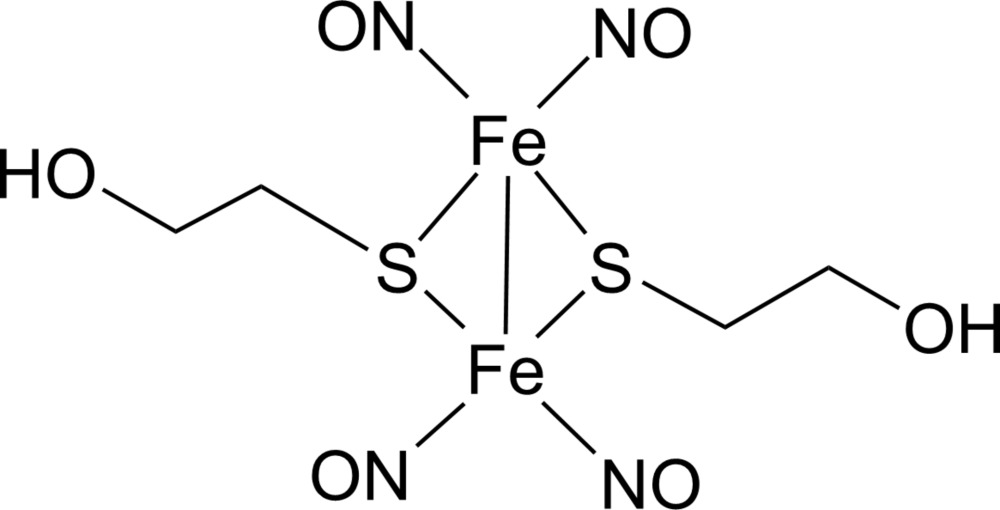


## Experimental

### 

#### Crystal data

[Fe_2_(C_2_H_5_OS)_2_(NO)_4_]
*M* *_r_* = 385.98
Monoclinic, 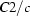

*a* = 16.943 (3) Å
*b* = 5.0070 (7) Å
*c* = 14.931 (2) Å
β = 94.327 (3)°
*V* = 1263.1 (3) Å^3^
*Z* = 4
Mo *K*α radiation
μ = 2.65 mm^−1^
*T* = 150 K
0.21 × 0.18 × 0.02 mm

#### Data collection

Bruker SMART APEXII diffractometer
Absorption correction: multi-scan (*SADABS*; Sheldrick, 1996 ▶) *T*_min_ = 0.606, *T*_max_ = 0.949
5708 measured reflections
1597 independent reflections
1240 reflections with *I* > 2σ
*R*_int_ = 0.039

#### Refinement

*R*[*F* ^2^ > 2σ(*F* ^2^)] = 0.028
*wR*(*F* ^2^) = 0.061
*S* = 0.94
1597 reflections
83 parameters
H-atom parameters constrained
Δρ_max_ = 0.52 e Å^−3^
Δρ_min_ = −0.32 e Å^−3^

### 

Data collection: *APEX2* (Bruker, 2007 ▶); cell refinement: *SAINT* (Bruker, 2007 ▶); data reduction: *SAINT*; program(s) used to solve structure: *SHELXTL* (Sheldrick, 2008 ▶); program(s) used to refine structure: *SHELXTL*; molecular graphics: *SHELXTL*; software used to prepare material for publication: *DIAMOND* (Brandenburg, 1999 ▶).

## Supplementary Material

Crystal structure: contains datablocks I, global. DOI: 10.1107/S1600536809048065/lh2939sup1.cif

Structure factors: contains datablocks I. DOI: 10.1107/S1600536809048065/lh2939Isup2.hkl

Additional supplementary materials:  crystallographic information; 3D view; checkCIF report

## Figures and Tables

**Table:** 

Fe1—N1	1.6650 (19)
Fe1—S1^i^	2.2555 (7)
Fe1—S1	2.2619 (6)
Fe1—Fe1^i^	2.7051 (6)

**Table:** 

N1—Fe1—N2	117.44 (9)
N1—Fe1—S1	110.22 (6)
N2—Fe1—S1	106.00 (7)

Symmetry code: (i) 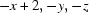.

**Table 2:** Hydrogen-bond geometry (Å, °)

*D*—H⋯*A*	*D*—H	H⋯*A*	*D*⋯*A*	*D*—H⋯*A*
O3—H3⋯O3^ii^	0.84	1.97	2.7950 (14)	166

Symmetry code: (ii) 
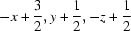
.

**Fig. 1.**
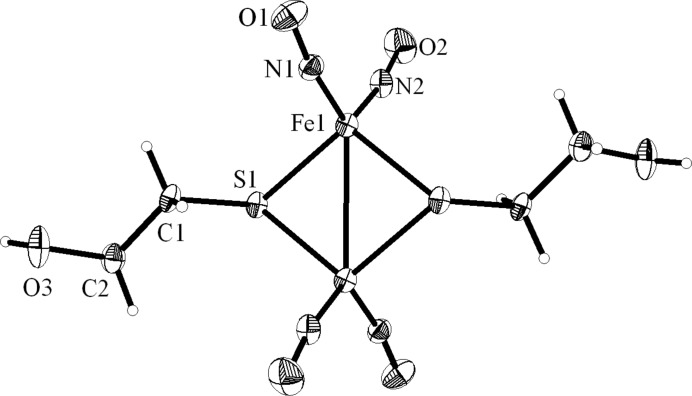
The molecular structure of the title complex, showing 50% displacement ellipsoids for non-H atoms. The H atoms are depicted by circles of an arbitrary radius. Unlabeled atoms of the complex are related to labeled atoms by the symmetry operator (2 - x, -y, - z).

**Fig. 2.**
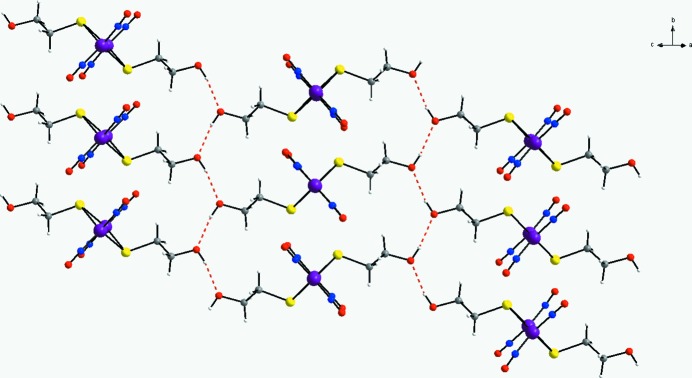
Part of the crystal structure showing intermolecular hydrogen bonds as dashed lines.

## supplementary crystallographic information

### Comment

Roussin's red esters (RREs) are photochemically active NO-release compounds and are expected to become potential NO donor drugs (Dillinger *et al.* 2007). The title compound is an air-stable and water soluble RRE compound. The molecular structure of the title complex is shown in Fig.1. The molecule lies on a crystallogrphic inversion center. The Fe-Fe distance is characteristic of a M-M bond (Dillinger *et al.* 2007). In the crystal structure, intermolecular O-H···O hydrogen bonds link complex molecules into a two-dimensional network.

### Experimental

FeSO_4_.7H_2_O (5.0 g, 0.018 mol) was added to H_2_SO_4_ (4*M*, 50 ml) at 273K. To the resulting solution, the mixture of NaNO_2_ (2.0 g, 0.023 mol) and mercaptoethanol (5.0 ml, 0.071 mol) were added. After stirring for 10 min, the solution was kept in refrigerator (277K) for one week. Dark red crystals formed and one was used for this structure determination. Yield: 2.1 g (65%). FTIR (THF): 1809 (*w*), 1774 (*vs*), 1748 (*s*) cm^-1 ^(νNO).

### Refinement

All H atoms were positioned geometrically and refined as riding atoms, with C_methylene_—H = 0.99, O—H = 0.84 Å while *U*_iso_(H) = 1.2*U*_eq_(C) and *U*_iso_(H) = 1.5*U*_eq_(O) for all the H atoms.

### Crystal data

**Table:**

[Fe_2_(C_2_H_5_OS)_2_(NO)_4_]	*F*(000) = 776
*M**_r_* = 385.98	*D*_x_ = 2.030 Mg m^−^^3^
Monoclinic, *C*2/*c*	Mo *K*α radiation, λ = 0.71073 Å
Hall symbol: -C 2yc	Cell parameters from 1286 reflections
*a* = 16.943 (3) Å	θ = 2.4–26.8°
*b* = 5.0070 (7) Å	µ = 2.65 mm^−^^1^
*c* = 14.931 (2) Å	*T* = 150 K
β = 94.327 (3)°	Plate, brown
*V* = 1263.1 (3) Å^3^	0.21 × 0.18 × 0.02 mm
*Z* = 4	

### Data collection

**Table:**

Bruker SMART APEXII diffractometer	1597 independent reflections
Radiation source: fine-focus sealed tube	1240 reflections with *I* > 2σ
graphite	*R*_int_ = 0.039
ω scans	θ_max_ = 28.7°, θ_min_ = 2.4°
Absorption correction: multi-scan (*SADABS*; Sheldrick, 1996)	*h* = −22→22
*T*_min_ = 0.606, *T*_max_ = 0.949	*k* = −6→6
5708 measured reflections	*l* = −19→19

### Refinement

**Table:**

Refinement on *F*^2^	Primary atom site location: structure-invariant direct methods
Least-squares matrix: full	Secondary atom site location: difference Fourier map
*R*[*F*^2^ > 2σ(*F*^2^)] = 0.028	Hydrogen site location: inferred from neighbouring sites
*wR*(*F*^2^) = 0.061	H-atom parameters constrained
*S* = 0.94	*w* = 1/[σ^2^(*F*_o_^2^) + (0.0324*P*)^2^] where *P* = (*F*_o_^2^ + 2*F*_c_^2^)/3
1597 reflections	(Δ/σ)_max_ = 0.003
83 parameters	Δρ_max_ = 0.52 e Å^−^^3^
0 restraints	Δρ_min_ = −0.32 e Å^−^^3^

### Special details

**Table:**

Geometry. All e.s.d.'s (except the e.s.d. in the dihedral angle between two l.s. planes) are estimated using the full covariance matrix. The cell e.s.d.'s are taken into account individually in the estimation of e.s.d.'s in distances, angles and torsion angles; correlations between e.s.d.'s in cell parameters are only used when they are defined by crystal symmetry. An approximate (isotropic) treatment of cell e.s.d.'s is used for estimating e.s.d.'s involving l.s. planes.
Refinement. Refinement of *F*^2^ against ALL reflections. The weighted *R*-factor *wR* and goodness of fit *S* are based on *F*^2^, conventional *R*-factors *R* are based on *F*, with *F* set to zero for negative *F*^2^. The threshold expression of *F*^2^ > σ(*F*^2^) is used only for calculating *R*-factors(gt) *etc*. and is not relevant to the choice of reflections for refinement. *R*-factors based on *F*^2^ are statistically about twice as large as those based on *F*, and *R*- factors based on ALL data will be even larger.

### Fractional atomic coordinates and isotropic or equivalent isotropic displacement parameters (Å^2^)

**Table:**

	*x*	*y*	*z*	*U*_iso_*/*U*_eq_	
C1	0.89173 (13)	0.1311 (4)	0.14871 (15)	0.0189 (5)	
H1A	0.8810	−0.0613	0.1385	0.023*	
H1B	0.9190	0.1523	0.2093	0.023*	
C2	0.81447 (12)	0.2848 (5)	0.14308 (15)	0.0219 (5)	
H2A	0.8253	0.4791	0.1449	0.026*	
H2B	0.7836	0.2436	0.0858	0.026*	
Fe1	1.062332 (16)	−0.00956 (6)	0.06209 (2)	0.01677 (10)	
N1	1.06466 (10)	−0.2257 (4)	0.14697 (12)	0.0200 (4)	
N2	1.13967 (11)	0.1950 (4)	0.05687 (13)	0.0216 (4)	
O1	1.07764 (10)	−0.3658 (3)	0.20944 (11)	0.0306 (4)	
O2	1.19777 (10)	0.3213 (4)	0.06634 (13)	0.0370 (5)	
O3	0.77015 (10)	0.2112 (3)	0.21691 (12)	0.0302 (4)	
H3	0.7591	0.3487	0.2456	0.045*	
S1	0.95465 (3)	0.25677 (10)	0.06417 (4)	0.01729 (13)	

### Atomic displacement parameters (Å^2^)

**Table:**

	*U*^11^	*U*^22^	*U*^33^	*U*^12^	*U*^13^	*U*^23^
C1	0.0256 (11)	0.0179 (11)	0.0142 (12)	−0.0014 (9)	0.0070 (8)	0.0007 (9)
C2	0.0243 (11)	0.0202 (12)	0.0220 (13)	−0.0032 (9)	0.0071 (9)	0.0006 (10)
Fe1	0.01897 (16)	0.01530 (17)	0.01622 (17)	−0.00062 (13)	0.00241 (11)	−0.00012 (13)
N1	0.0220 (9)	0.0206 (10)	0.0173 (10)	−0.0001 (8)	0.0012 (7)	−0.0025 (8)
N2	0.0225 (9)	0.0206 (10)	0.0224 (11)	−0.0002 (8)	0.0060 (8)	−0.0021 (8)
O1	0.0449 (10)	0.0241 (9)	0.0220 (10)	−0.0008 (8)	−0.0020 (8)	0.0069 (7)
O2	0.0276 (9)	0.0363 (11)	0.0477 (12)	−0.0119 (8)	0.0069 (8)	−0.0058 (9)
O3	0.0378 (9)	0.0235 (9)	0.0322 (11)	−0.0049 (8)	0.0222 (8)	−0.0037 (7)
S1	0.0212 (2)	0.0139 (3)	0.0174 (3)	−0.0006 (2)	0.00546 (19)	−0.0004 (2)

### Geometric parameters (Å, °)

**Table:**

C1—C2	1.515 (3)	Fe1—N2	1.6696 (19)
C1—S1	1.824 (2)	Fe1—S1^i^	2.2555 (7)
C1—H1A	0.9900	Fe1—S1	2.2619 (6)
C1—H1B	0.9900	Fe1—Fe1^i^	2.7051 (6)
C2—O3	1.428 (2)	N1—O1	1.174 (2)
C2—H2A	0.9900	N2—O2	1.170 (2)
C2—H2B	0.9900	O3—H3	0.8400
Fe1—N1	1.6650 (19)	S1—Fe1^i^	2.2555 (7)
			
C2—C1—S1	109.51 (15)	N2—Fe1—S1^i^	110.41 (7)
C2—C1—H1A	109.8	N1—Fe1—S1	110.22 (6)
S1—C1—H1A	109.8	N2—Fe1—S1	106.00 (7)
C2—C1—H1B	109.8	S1^i^—Fe1—S1	106.43 (2)
S1—C1—H1B	109.8	N1—Fe1—Fe1^i^	121.14 (6)
H1A—C1—H1B	108.2	N2—Fe1—Fe1^i^	121.42 (7)
O3—C2—C1	109.18 (18)	S1^i^—Fe1—Fe1^i^	53.324 (17)
O3—C2—H2A	109.8	S1—Fe1—Fe1^i^	53.106 (18)
C1—C2—H2A	109.8	O1—N1—Fe1	170.09 (16)
O3—C2—H2B	109.8	O2—N2—Fe1	169.24 (18)
C1—C2—H2B	109.8	C2—O3—H3	109.5
H2A—C2—H2B	108.3	C1—S1—Fe1^i^	110.16 (7)
N1—Fe1—N2	117.44 (9)	C1—S1—Fe1	108.68 (7)
N1—Fe1—S1^i^	105.88 (7)	Fe1^i^—S1—Fe1	73.57 (2)

Symmetry codes: (i) −*x*+2, −*y*, −*z*.

### Hydrogen-bond geometry (Å, °)

**Table:**

*D*—H···*A*	*D*—H	H···*A*	*D*···*A*	*D*—H···*A*
O3—H3···O3^ii^	0.84	1.97	2.7950 (14)	166

Symmetry codes: (ii) −*x*+3/2, *y*+1/2, −*z*+1/2.
